# Supplementary material for: Empirical methods for controlling false positives and estimating confidence in ChIP-Seq peaks
Source: BMC Bioinformatics. 2008 Dec 5;9:523. doi: 10.1186/1471-2105-9-523 (PMC2628906; doi:10.1186/1471-2105-9-523)
Supplement: Additional File 1 — A variety of html documents from the USeq web site detailing the available applications, their best usage, output file type descriptions, command line menus, etc. [file 1471-2105-9-523-S1.zip › USeqUserGuides/JavaDoc.zip!/JavaDoc/util/bio/wrappers/Segment.html]

Segment


|  |  |  |
| --- | --- | --- |
| **Overview** \| **Package** \| **Class** \| **Use** \| **Tree** \| **Deprecated** \| **Index** \| **Help** | |  |
| **PREV CLASS**   **NEXT CLASS** | **FRAMES**    **NO FRAMES**     **All Classes** |  |
| SUMMARY: NESTED \| FIELD \| CONSTR \| METHOD | DETAIL: FIELD \| CONSTR \| METHOD |  |


---


## util.bio.wrappers Class Segment

```
java.lang.Object
  util.bio.wrappers.Segment
```

---

``` public class Segment extends java.lang.Object ```

This wraps Huber's segment() function in his 'tilingArray' R package.
Use it to divide each column of float[]s, in a tab delimited file, into 3 segments, 2 indexes.
The first row in the file should contain the number of subsequent floats in each column to use during segmentation.
Must have installed the BioConductor tilingArray package and it's 1/2 dozen or so dependancies.

---

**Constructor Summary**

`Segment(java.io.File rApp,
java.io.File dataFile,
java.io.File tempDirectory)`


| **Method Summary** | |
| --- | --- |
| `void` | `checkFiles()` |
| `void` | `deleteTempFiles()` |
| `void` | `executeRScript()` |
| `int[][]` | `getSegmentIndexes()` |
| `void` | `parseResults()` |
| `void` | `segment()` |
| `void` | `writeRScriptFile()` |

**Methods inherited from class java.lang.Object**

`equals, getClass, hashCode, notify, notifyAll, toString, wait, wait, wait`

**Constructor Detail**

### Segment

```
public Segment(java.io.File rApp,
               java.io.File dataFile,
               java.io.File tempDirectory)
```


**Method Detail**

### segment

```
public void segment()
             throws java.lang.Exception
```

:   **Throws:**: `java.lang.Exception`

---


### deleteTempFiles

```
public void deleteTempFiles()
```

---


### parseResults

```
public void parseResults()
                  throws java.lang.Exception
```

:   **Throws:**: `java.lang.Exception`

---


### executeRScript

```
public void executeRScript()
                    throws java.lang.Exception
```

:   **Throws:**: `java.lang.Exception`

---


### checkFiles

```
public void checkFiles()
                throws java.lang.Exception
```

:   **Throws:**: `java.lang.Exception`

---


### writeRScriptFile

```
public void writeRScriptFile()
                      throws java.lang.Exception
```

:   **Throws:**: `java.lang.Exception`

---


### getSegmentIndexes

```
public int[][] getSegmentIndexes()
```


---


|  |  |  |
| --- | --- | --- |
| **Overview** \| **Package** \| **Class** \| **Use** \| **Tree** \| **Deprecated** \| **Index** \| **Help** | |  |
| **PREV CLASS**   **NEXT CLASS** | **FRAMES**    **NO FRAMES**     **All Classes** |  |
| SUMMARY: NESTED \| FIELD \| CONSTR \| METHOD | DETAIL: FIELD \| CONSTR \| METHOD |  |


---
